# Integrating Transcriptomics and Machine Learning to Uncover the FLI1-PARP14-Immune Axis in Ulcerative Colitis Activity and Pathogenesis

**DOI:** 10.3390/genes16111342

**Published:** 2025-11-07

**Authors:** Zhizhong Zheng, Yayu Zhang, Zhixing Gao, Houyu Chen, Gang Song

**Affiliations:** Cancer Research Center, School of Medicine, Xiamen University, Xiamen 361102, China; zhizhong29@163.com (Z.Z.); z17786185903@163.com (Y.Z.); gaozhixing@stu.xmu.edu.cn (Z.G.); c283314665@163.com (H.C.)

**Keywords:** ulcerative colitis, diagnostic biomarkers, *PARP14*, machine learning, immune infiltration

## Abstract

**Background:** Ulcerative colitis (UC) is a chronic inflammatory bowel disease whose molecular mechanisms of action remain incompletely characterized. This study was designed to develop potential diagnostic biomarkers and unravel the pathogenic causes of UC activity through the integration of transcriptome analysis with machine learning and genetic causal inference. **Methods:** Gene expression datasets (GSE75214, GSE53306, GSE179285) from the GEO database were evaluated. Weighted gene co-expression network analysis (WGCNA) and differentially expressed gene (DEG) analysis were applied to discover activity-associated genes. Protein–protein interaction networks and ensemble machine learning methods were utilized to refine the potential list. Furthermore, summary-data-based Mendelian Randomization (SMR) analysis and immune infiltration research were conducted. **Results:** Eight characteristic genes were identified, with *CXCL11*, *PARP14*, and *IFITM1* emerging as hub genes. These hub genes exhibited strong diagnostic accuracy, with consistent area under the curve (AUC) values exceeding 0.83 across 3 independent cohorts. SMR analysis demonstrated a probable causal connection between higher *PARP14* and UC susceptibility. The hub genes were strongly correlated with immune cells, including M1 macrophages and NK cells. *FLI1* was discovered as a critical upstream transcription factor regulating this network. **Conclusions:** The findings outline a *FLI1-PARP14*-immune axis central to UC activity, providing unique insights into its pathophysiology and highlighting *PARP14* as a promising diagnostic biomarker and potential therapeutic target.

## 1. Introduction

Inflammatory bowel disease (IBD) is a group of chronic, relapsing inflammatory disorders of the gastrointestinal tract. It is characterized by recurrent mucosal inflammation, which can result in irreversible tissue damage, impaired intestinal function, and systemic complications such as malnutrition and extraintestinal manifestations [1]. Among its 2 major subtypes, ulcerative colitis (UC) is characterized by diffuse, continuous mucosal inflammation that originates in the rectum and progresses proximally across the colon. In contrast, Crohn’s disease (CD) can affect any segment of the gastrointestinal system and often manifests with discontinuous lesions [2,3]. The global burden of UC has risen considerably over recent decades. By 2023, its prevalence was anticipated at roughly 5 million cases worldwide, with incidence growing in both developed and developing regions. This trend, driven by the combination of genetic susceptibility, Westernized diets, microbial dysbiosis, and environmental exposures, imposes major physical, psychological, and economic costs on patients and healthcare systems [3,4].

The pathophysiology of UC is multifaceted and not yet fully elucidated. It involves complex interactions among genetic predisposition, intestinal barrier dysfunction, immunological dysregulation, and alterations in the gut microbiota [5,6]. Genome-wide association studies (GWAS) have identified more than 200 susceptibility loci, many of which are associated with pathways critical to epithelial integrity (e.g., MUC2-mediated mucus barrier), immune regulation (e.g., TNF-α and IL-23 signaling), and microbial recognition (e.g., *NOD2*) [7,8]. Environmental risk factors, such as early antibiotic exposure, consumption of processed foods, and persistent psychological stress, further alter intestinal homeostasis by reducing microbial diversity (e.g., decreased Bacteroidetes and increased Proteobacteria) and increasing epithelial permeability. This disturbance contributes to the development of a “leaky gut”, which, in turn, can worsen mucosal inflammation [6,9]. Despite the considerable progress in the field of UC research, the molecular pathways underlying its development, relapse, and progression remain poorly characterized. This limitation, in turn, constrains the efficacy of current therapeutic options.

Current management strategies for UC reflect its incurable nature and focus on 4 key goals: inducing a rapid clinical response, maintaining long-term remission, promoting mucosal healing, and preventing complications such as colorectal cancer and toxic megacolon [10,11]. Therapeutic regimens are stratified by disease severity and extent. Meta-analyses demonstrate the efficacy of 5-aminosalicylic acid (5-ASA) over placebo in reducing mucosal inflammation and relapse rates [12]. For mild-to-moderate UC, 5-ASA formulations, taken orally or rectally, remain the primary therapy for inducing and maintaining remission. For patients with moderate-to-severe disease who do not respond adequately to conventional treatments, the therapeutic landscape has expanded significantly to include biologic agents, particularly inhibitors targeting specific biomarkers [13]. While systemic corticosteroids remain highly effective for short-term symptom relief in moderate-to-severe UC, their use is restricted to acute settings due to significant long-term adverse effects, including osteoporosis and increased infection risk [14].

A critical challenge in UC management is the absence of individualized strategies to anticipate relapse, maximize therapeutic response, and achieve lasting mucosal healing—essential criteria for improving long-term outcomes and quality of life [10,15]. Mucosal healing, defined as the endoscopic normalization of the colonic mucosa, is a critical therapeutic target that is correlated with lower hospitalization and colectomy rates, as well as a reduced risk of colorectal cancer [10,11]. This is particularly relevant given that the risk of colorectal cancer for any patient with ulcerative colitis is known to be elevated, and is estimated to be 2% after 10 years, 8% after 20 years and 18% after 30 years of disease [16].

However, traditional biomarkers (e.g., fecal calprotectin and C-reactive protein) and clinical symptom scores often fail to detect the subtle molecular changes that drive disease activity or remission [17,18]. Histology, while a more definitive tool for diagnosing disease activity and severity and featuring higher accessibility in clinical practice, also struggles to rapidly capture these early subtle molecular alterations. In contrast, transcriptomics, though less accessible compared to histology, demonstrates advantages in quickly reflecting such fine-grained molecular changes in the early stages of disease.

This complementary nature of the two methods highlights that their integration could better address existing diagnostic gaps—an approach that further underscores the urgent need for diagnostic strategies capable of rapidly reflecting treatment efficacy. Such combined strategies, leveraging both histology’s diagnostic definitiveness and transcriptomics’ molecular sensitivity, would enable timely intervention and improved personalized management, while also helping identify molecular fingerprints to distinguish active from inactive disease phases and uncover key regulatory genes involved in inflammation resolution, epithelial repair, and immune homeostasis.

Recent advancements in high-throughput sequencing and analytics have enabled systematic analysis of transcriptome profiles between inactive and active disease phases. Differential gene expression studies of patient samples have identified potential diagnostic biomarkers such as *CCL11* and *MMP1*, which contribute to immune cell recruitment and extracellular matrix remodeling—processes implicated in mucosal inflammation and healing [18]. Weighted gene co-expression network analysis (WGCNA) and machine learning algorithms have further defined important hub genes, including *CTSS*, *S100A11*, and *TUBB*, which are dysregulated in UC and linked to macrophage-mediated inflammation and epithelial barrier dysfunction [19]. Integrating these results with expression quantitative trait loci (eQTL) analysis [20] and Mendelian randomization [21] offers a means to establish causal relationships between gene expression and UC susceptibility, while protein–protein interaction (PPI) networks and immune cell infiltration analysis (e.g., CIBERSORT) provide mechanistic insights into how hub genes regulate inflammatory pathways and immune cell dynamics [7,18,22].

By comprehensively comparing gene expression profiles between inactive and active disease phases, this study aims to discover hub genes driving UC activity. The overarching goal is to facilitate the development of targeted therapeutics capable of inducing durable remission, promoting mucosal healing, and improving patient quality of life. These discoveries are expected to address important gaps in understanding the molecular mechanisms of UC and advance the development of precision medicine strategies for this complex disease.

## 2. Materials and Methods

### 2.1. Study Design

**Figure 1:**
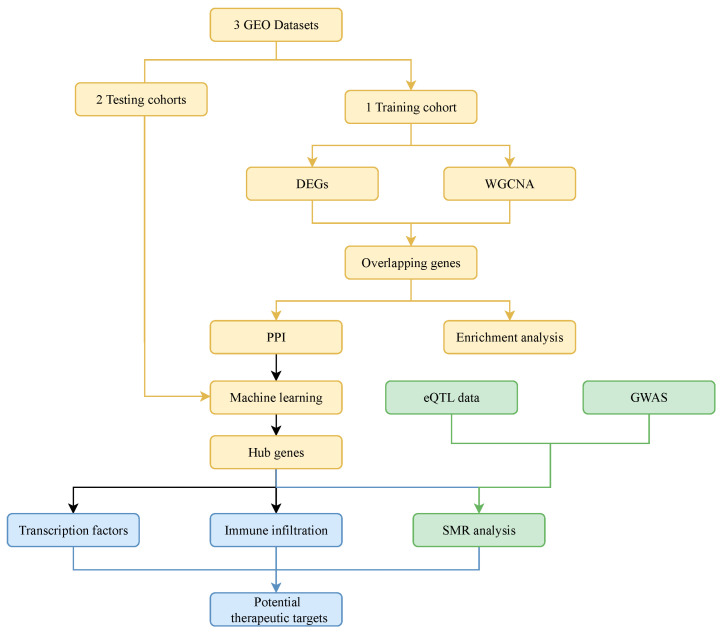
Flowchart of the study. Abbreviations: gene expression omnibus (GEO); differentially expressed genes (DEGs); weighted gene co-expression network analysis (WGCNA); protein–protein interaction (PPI); expression quantitative trait locus (eQTL); genome-wide association study (GWAS); summary-data-based mendelian randomization (SMR).

Using the GEO database [23], overlapping genes associated with UC activity were identified through weighted gene co-expression network analysis (WGCNA) and differentially expressed gene (DEG) analysis. Characteristic and hub genes were further evaluated using protein–protein interaction (PPI) analysis and machine learning algorithms. Immune infiltration patterns associated with UC activity, together with hub genes were also investigated. In addition, expression quantitative trait locus (eQTL) data and genome-wide association study (GWAS) data related to IBD were integrated to examine causal links between hub genes and IBD. The overall analytical workflow is depicted in Figure 1.

### 2.2. Data Collection and Analysis

Three gene expression profiles (GSE75214 [24], GSE53306 [25], and GSE179285 [26]) of UC patients were retrieved from the GEO database (https://www.ncbi.nlm.nih.gov/geo/ (accessed on 5 August 2025)), maintained by the National Center for Biotechnology Information (NCBI), Bethesda, MD, USA. The GSE75214 dataset was used for DEG analysis and selected as the training cohort. The GSE53306 and GSE179285 datasets were deployed as independent testing cohorts (Table 1).

### 2.3. WGCNA

To construct a weighted gene co-expression network from the top 5000 most variably expressed genes, the R package “WGCNA” (version 1.73; https://cran.r-project.org/web/packages/WGCNA/index.html (accessed on 5 August 2025)) [27] was utilized to identify gene modules strongly correlated with UC activity. A scale-free network was built by determining the appropriate soft-thresholding power (β). A weighted adjacency matrix was constructed and subsequently transformed into a topological overlap matrix (TOM). Hierarchical clustering was then performed using a TOM-based dissimilarity metric. Gene modules were identified by dynamic tree cutting, and genes significantly correlated with UC activity were selected for further analysis.

### 2.4. Functional Enrichment Analysis

Functional enrichment analyses were performed on the overlapping genes of the DEGs and the UC activity-associated module to uncover their biological roles. Gene Ontology (GO) and Kyoto Encyclopedia of Genes and Genomes (KEGG) enrichment analysis was performed using the R packages “clusterProfiler” and “DOSE” (version 4.2.0), supplemented by the org.Hs.eg.db database.

### 2.5. Protein–Protein Interaction (PPI) Analysis

A PPI network was constructed using the STRING database (https://string-db.org/ (accessed on 6 August 2025)) [20], focusing on genes overlapping between the DEGs and the UC activity-associated module. The network was visualized using Cytoscape software (version 3.10.2). To identify characteristic genes, the cytoHubba plugin was used to extract the top 15 genes with the highest maximal clique centrality (MCC) scores, emphasizing functionally significant sub-networks.

### 2.6. Machine Learning Algorithm Analysis

The top 15 genes identified from the PPI analysis were assessed using a diverse set of machine learning algorithms to identify the most predictive features. Twelve algorithms were implemented, including regularization methods such as Least Absolute Shrinkage and Selection Operator (Lasso), Ridge Regression (Ridge), and Elastic Net (ElasticNet); generalized linear models such as Stepwise Generalized Linear Model (Stepglm), Boosted Generalized Linear Model (glmBoost), and Partial Least Squares Regression with Generalized Linear Model (plsRglm); ensemble learners such as Random Forest (RF), Gradient Boosting Machine (GBM), and Extreme Gradient Boosting (XGBoost); and pattern recognition approaches such as Support Vector Machine (SVM), Linear Discriminant Analysis (LDA), and Naive Bayes Classifier (Naive Bayes) [28]. This integrative strategy ensured coverage of both linear and nonlinear modeling paradigms.

The computational approach involved 2 stages: (1) feature ranking by recursive feature elimination for preliminary screening and (2) predictive modeling through stacked generalization. All models were trained with stratified 10-fold cross-validation and underwent systematic hyperparameter optimization, resulting in 113 unique configurations. Four algorithms with intrinsic feature selection ability (Lasso, Random Forest, Stepglm, and glmBoost) were initially utilized to refine the gene set. The refined subsets were subsequently used to train 8 additional algorithms for predictive modeling. Each of the 113 models was evaluated based on the area under the receiver operating characteristic curve (AUC) in both training and testing cohorts. AUC values close to 1 were considered to indicate strong discriminative performance, whereas values near 0.5 suggested no diagnostic utility. The optimum model was selected based on the highest mean AUC across validation folds.

### 2.7. Diagnostic Value and Expression Patterns of Hub Genes

To investigate the diagnostic potential of hub genes in UC activity, receiver operating characteristic (ROC) curve analysis was performed using the R package “pROC” (version 1.19.0.1). The AUC was used to assess each gene’s ability to differentiate between inactive and active phase. Genes having AUC values greater than 0.7 in both the training and testing cohorts were identified as hub genes with biomarker potential. Furthermore, a logistic regression model was developed using the R package “glmnet” (version 4.1-10) to analyze the combined diagnostic performance of these hub genes in both the training (GSE75214) and testing (GSE179285) cohorts.

### 2.8. Prediction of Transcription Factors

To investigate upstream regulatory mechanisms of hub genes, the TF Target Finder (TFTF) database (https://jingle.shinyapps.io/TF_Target_Finder/ (accessed on 6 August 2025)) [29] was used to predict transcription factors (TFs) potentially targeting these genes. It was hypothesized that TFs, upregulated in UC, may actively influence hub gene expression. Common TFs among the 8 characteristic genes were identified by petal diagram analysis. A TF–gene regulation network was subsequently built, and functionally relevant sub-networks were extracted.

### 2.9. Summary-Data–Based Mendelian Randomization (SMR) Analysis

To evaluate potential causal relationships between gene expression and UC susceptibility, summary-data–based Mendelian randomization (SMR) analysis [30] was conducted. The analysis focused on cis-regions and incorporated heterogeneity in dependent instruments (HEIDI) tests to assess instrumental variable heterogeneity and minimize horizontal pleiotropy. Genetic instruments were derived from single nucleotide polymorphisms (SNPs), integrating eQTLs as exposures and GWAS summary statistics for IBD from the FinnGen biobank (version R12; https://r12.finngen.fi/ (accessed on 7 August 2025)).

### 2.10. Immune Cell Infiltration Analysis

To assess immune cell dynamics in UC, CIBERSORT analysis was performed, a deconvolution method based on linear support vector regression that infers immune cell composition from bulk gene expression data. The Leukocyte Signature Matrix (LM22) was utilized as a reference to quantify the relative abundances of 22 immune cell types (CIBERSORTx; https://cibersortx.stanford.edu/ (accessed on 7 August 2025)) [31]. Correlation analysis was then performed between hub gene expression and immune cell subtypes to identify relationships relevant to UC activity.

### 2.11. Statistical Analysis

All statistical analyses were performed using R software (version 4.5.1). Pearson correlation coefficients were calculated to examine hub gene expression patterns. Spearman’s rank correlation was utilized to analyze relationships between immune cell infiltration and hub gene transcriptional activity. A *p*-value < 0.05 was considered statistically significant.

## 3. Results

### 3.1. DEGs Correlated with UC Activity

**Figure 2:**
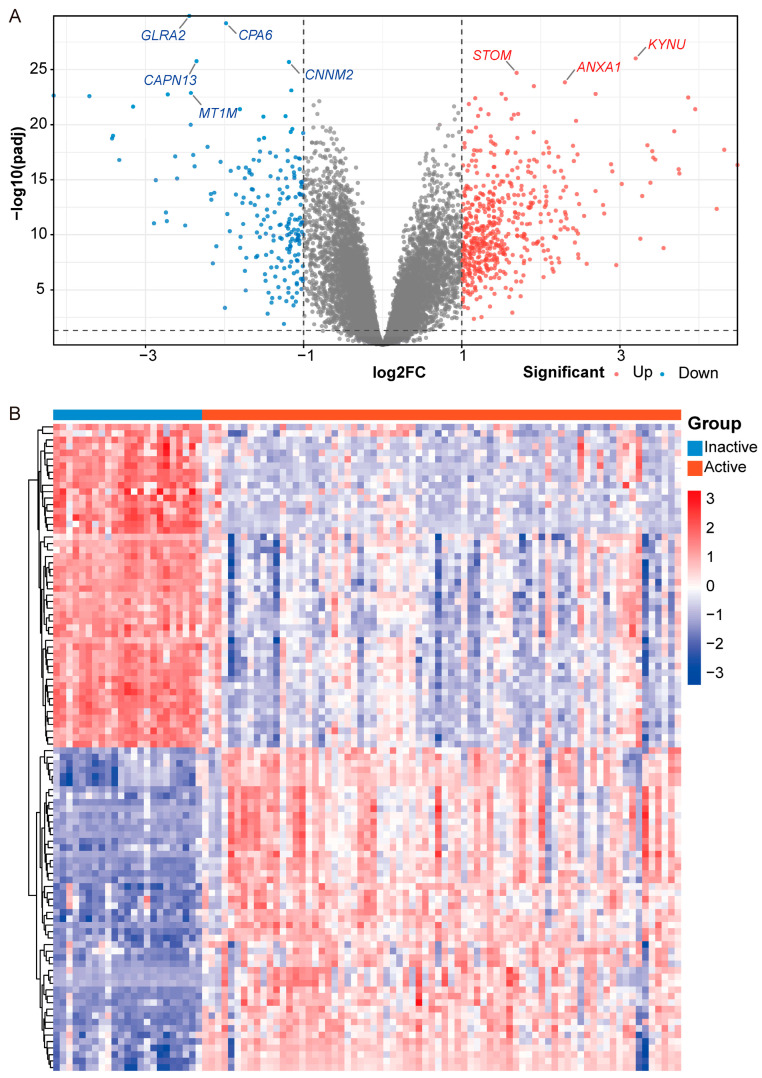
Differentially expressed genes analysis (DEGs) for UC activity in the GSE75214 dataset (*n* = 97; 23 inactive, 74 active). (**A**) Volcano plot of DEGs. Blue represents downregulated genes (log_2_FC < −1, FDR < 0.05), red represents upregulated genes (log_2_FC > 1, FDR < 0.05), and gray represents non-differentially expressed genes. (**B**) Clustered heatmap of the top 50 DEGs. Rows represent DEGs; columns represent samples.

To identify DEGs associated with disease activity in UC, the GSE75214 dataset was analyzed. Samples were classified into 2 groups based on UC activity: inactive phase and active phase. DEG analysis was performed using the limma package in R, with thresholds set at |log_2_FC| > 1 and false discovery rate (FDR) < 0.05. A total of 715 DEGs (194 Down and 521 Up) were identified between inactive and active phase (Figure 2A). The top 50 DEGs are visualized in a clustered heatmap (Figure 2B).

### 3.2. Meorange Module Strongest Correlated with UC Activity

**Figure 3:**
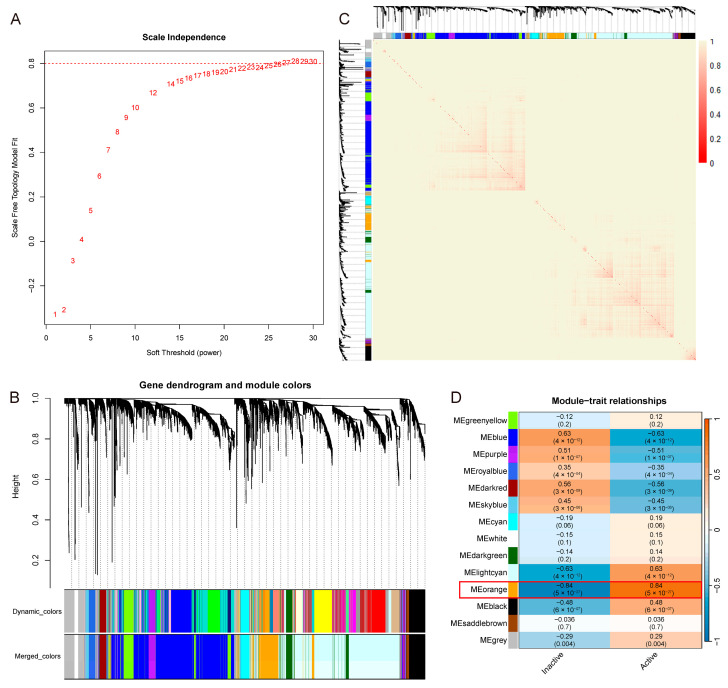
Weighted gene co-expression network analysis (WGCNA) results for UC activity. (**A**) Threshold selection for WGCNA analysis; the optimal soft-thresholding power was 28. (**B**) Gene clustering dendrogram generated by WGCNA. (**C**) Heatmap of co-expression patterns across merged gene modules. (**D**) Module–trait relationships. Each cell contains the Pearson correlation coefficient and corresponding *p*-value; the color gradient indicates the direction and strength of the correlation.

WGCNA was applied to the GSE75214 dataset to identify co-expression modules associated with UC activity. Soft-thresholding powers ranging from 1 to 30 were evaluated using the scale-free topology fit index and mean connectivity (Figure 3A). A power value of 28 was selected, achieving a scale-free fit over 0.8 while maintaining strong connectivity. Using this threshold, a co-expression network was constructed, and gene clusters were visualized in a dendrogram (Figure 3B). Modules identified with the dynamic tree cut algorithm were represented as color-coded branches, with correlated modules subsequently merged. A cluster heatmap of all genes demonstrated intermodular co-expression patterns, where color intensity reflected gene–gene connectivity strength (Figure 3C). Module–trait correlation analysis revealed that the Meorange module had the strongest positive correlation with active phase (R = 0.84, *p* < 0.05; Figure 3D). This module comprised 383 genes after merging.

### 3.3. Immune System and Cytokine-Driven Inflammation Closely Associated with UC Activity

**Figure 4:**
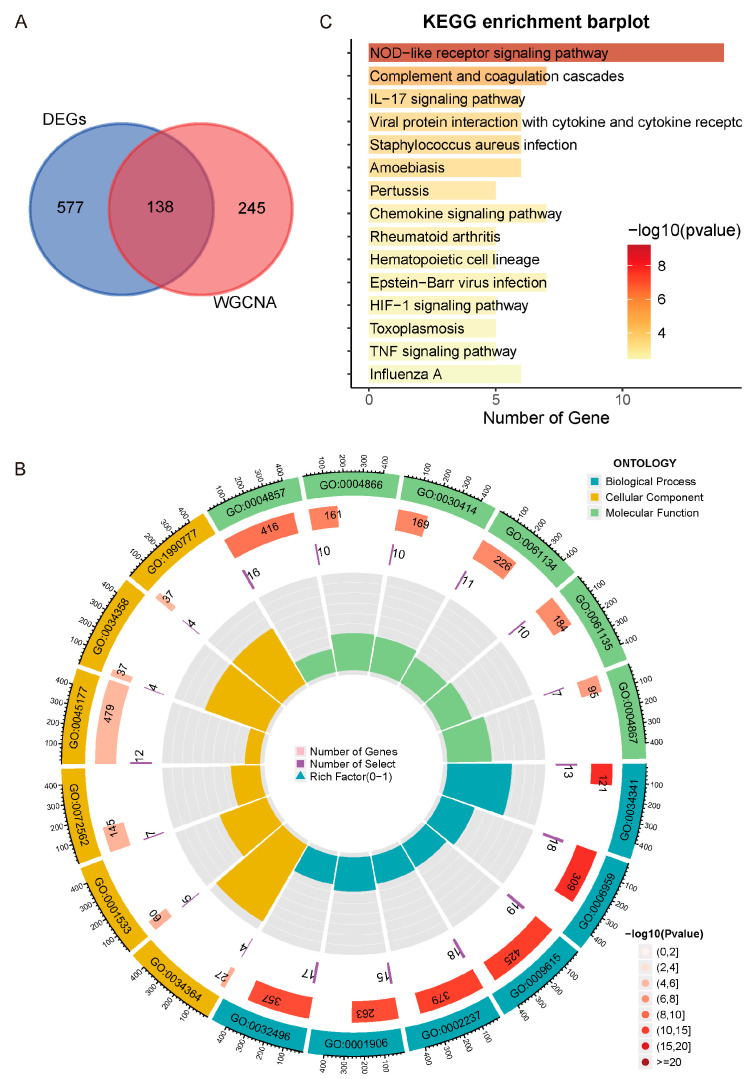
Functional enrichment analysis of genes overlapping between differentially expressed genes (DEGs) and the Weighted gene co-expression network analysis (WGCNA) -identified Meorange module. (**A**) Venn diagram illustrating the intersection of DEGs and Meorange module genes. (**B**) Gene ontology (GO) enrichment analysis: circle plot displaying the top significantly enriched terms. (**C**) Kyoto encyclopedia of genes and genomes (KEGG) pathway enrichment analysis: the top 15 significantly enriched pathways.

To elucidate molecular mechanisms underlying UC activity, genes overlapping the DEGs (*n* = 715) and the Meorange module (*n* = 383) were identified. A Venn diagram showed 138 intersecting genes (Figure 4A), suggesting candidates potentially driving UC activity.

These 138 genes were subjected to GO and KEGG enrichment analysis. GO analysis covered biological process (BP), molecular function (MF), and cellular component (CC) categories. Significantly enriched BP terms were primarily associated with immune regulation, including “response to type II interferon” (reflecting IFN-γ-mediated antiviral and pro-inflammatory responses), “humoral immune response” (antibody-mediated pathogen clearance), “response to virus” (viral recognition and defense), and “response to molecule of bacterial origin” (pattern recognition of microbial antigens) (Figure 4B).

KEGG pathway analysis further emphasized inflammatory and immune signaling, notably the “NOD-like receptor signaling pathway” (cytosolic pathogen sensing and inflammasome activation), “Complement and coagulation cascades” (innate immune amplification and vascular regulation), and the “IL-17 signaling pathway” (a critical mediator of mucosal inflammation in UC) (Figure 4C). Collectively, these results indicate that UC activity is closely associated with immune system and cytokine-driven inflammation, providing a biological rationale for targeting key disease pathways.

### 3.4. Characteristic Genes Associated with UC Activity

**Figure 5:**
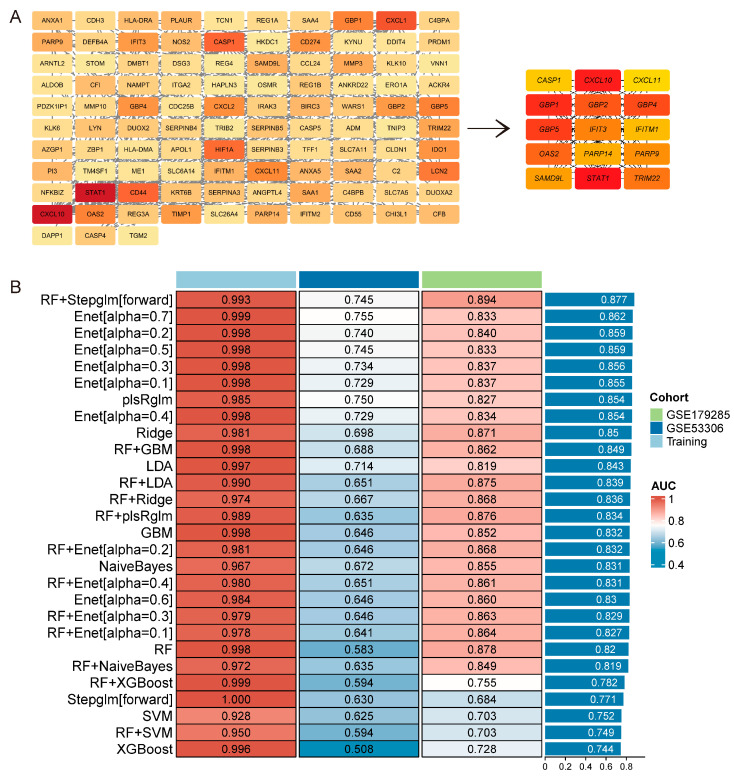
Protein–protein interaction (PPI) and Machine Learning analysis. (**A**) Top 15 genes identified from the PPI network. (**B**) Performance evaluation of 113 machine learning algorithm combinations using stratified 10-fold cross-validation. Abbreviations: Area under the receiver operating characteristic curve (AUC).

The top 15 genes identified from the PPI network (Figure 5A) were considered as pivotal regulators of UC-related biological processes. To refine these candidates, machine learning was applied, constructing 113 predictive models using combinations of algorithms. The combination of Random Forest and Stepglm [forward] achieved the highest mean AUC (0.877) in both training and testing cohorts (Figure 5B), demonstrating superior discriminative capacity. From this model, 8 characteristic genes were identified: *SAMD9L* (Sterile Alpha Motif Domain Containing 9 Like), *IFITM1* (Interferon-Induced Transmembrane Protein 1), *GBP5* (Guanylate Binding Protein 5), *PARP14* (Poly (ADP-Ribose) Polymerase 14), *PARP9* (Poly (ADP-Ribose) Polymerase 9), *CXCL10* (C-X-C Motif Chemokine Ligand 10), *GBP1* (Guanylate Binding Protein 1), and *CXCL11* (C-X-C Motif Chemokine Ligand 11). These genes represent promising candidates for further mechanistic studies and biomarker or therapeutic target development.

### 3.5. Hub Genes and Diagnostic Capability in UC Activity

**Figure 6:**
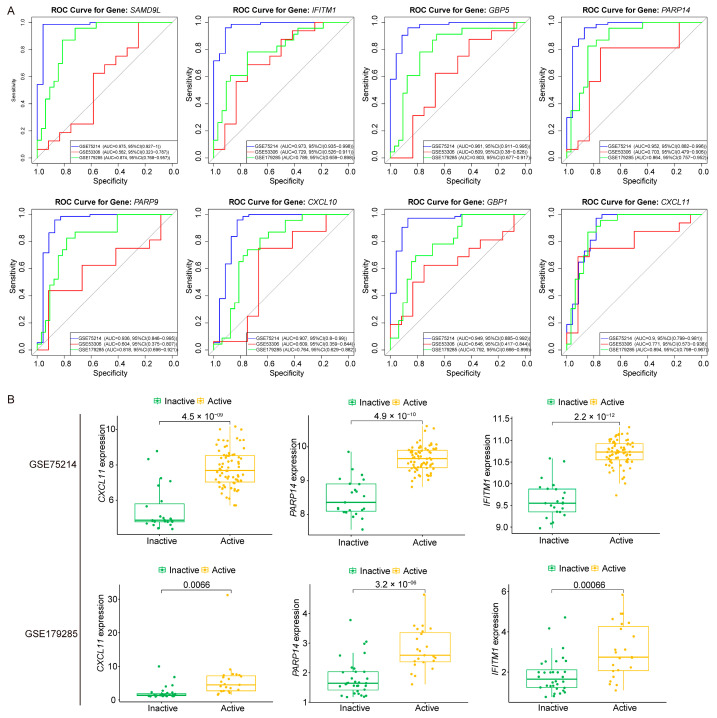
Diagnostic performance and expression validation of characteristic genes. (**A**) Receiver operating characteristic (ROC) curves of the 8 characteristic genes in the training and testing cohorts. (**B**) Expression levels of the 3 hub genes (*CXCL11*, *PARP14*, *IFITM1*) in the training and testing cohorts.

The diagnostic performance of the 8 characteristic genes was evaluated using the pROC package by performing ROC curve analysis in both training and testing cohorts. Among them, *CXCL11*, *PARP14*, and *IFITM1* consistently displayed high diagnostic accuracy, with AUC values surpassing 0.83 in both cohorts (Figure 6A, Table 2). The notable performance suggests their high sensitivity and specificity in discriminating active phase from inactive phase. Based on these results, *CXCL11*, *PARP14*, and *IFITM1* were identified as hub genes for UC activity.

Validation in 2 independent datasets confirmed their diagnostic potential. In GSE75214, all 3 genes were significantly upregulated in active phase compared to inactive phase (*p* < 0.05). Similarly, in GSE179285, they showed consistent overexpression in active phase (Figure 6B). Despite variations in sample collection, patient demographics, and technology platforms, their diagnostic performance remained consistent. These findings strongly support *CXCL11*, *PARP14*, and *IFITM1* as reliable diagnostic biomarkers for UC activity.

### 3.6. TFs of Characteristic Genes in UC Activity

**Figure 7:**
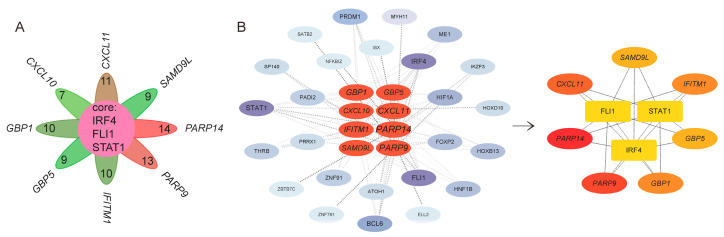
Transcriptional regulatory network analysis of characteristic genes. (**A**) Petal diagram of transcription factors (TFs) targeting the 8 characteristic genes. Each petal represents 1 gene (labeled with gene name), with the number of regulating TFs indicated in parentheses; the central region shows 3 common TFs (FLI1, STAT1, and IRF4) targeting all 8 genes. (**B**) Core TF–gene regulatory subnetwork, comprising 3 TFs and 7 characteristic genes, highlighting key transcriptional interactions in UC activity.

To investigate transcriptional regulation, upstream transcription factors (TFs) were predicted using the TFTF database. Each gene was regulated by multiple TFs: *CXCL11* (11), *CXCL10* (7), *GBP1* (10), *GBP5* (9), *IFITM1* (10), *PARP9* (13), *PARP14* (14), and *SAMD9L* (9). Petal diagram analysis identified 3 TFs common to all 8 genes: *FLI1, STAT1,* and *IRF4* (Figure 7A). A TF–gene regulatory network was constructed with Cytoscape and analyzed via cytoHubba, which confirmed the same 3 TFs but excluded *CXCL10* due to weaker regulatory connectivity (Figure 7B).

Expression validation in both training and testing datasets (Table 3) showed that (1) STAT1 and IRF4 lacked consistent differential expression, and (2) STAT1 displayed variable trends between cohorts. Consequently, only FLI1 was considered as a major TF regulating characteristic genes in UC activity.

### 3.7. Causal Association Between PARP14 Expression and UC Risk Identified by SMR Analysis

**Figure 8:**
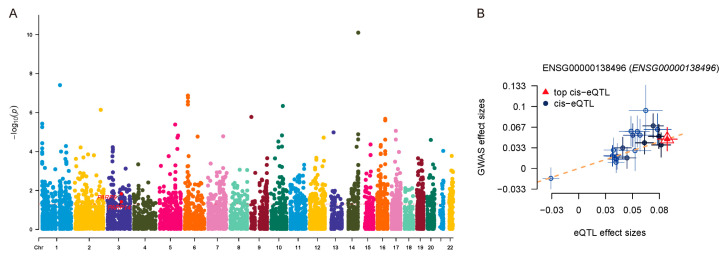
Summary-data–based mendelian randomization (SMR) analysis integrating expression quantitative trait locus (eQTL) and genome-wide association study (GWAS) data (FinnGen R12). (**A**) Manhattan plot illustrating genes associated with IBD susceptibility. (**B**) Scatter plot of eQTL versus GWAS effect sizes for the *PARP14* locus, indicating a shared genetic association.

SMR analysis integrating GTEx v8 eQTL data and FinnGen R12 IBD GWAS data identified a significant causal association between *PARP14* and UC susceptibility (Table 4). Among the 3 hub genes tested, only *PARP14* retained statistical significance following colocalization analysis (Figure 8A). The colocalization plot of GWAS and eQTL signals for *PARP14* showed clustering of effect sizes, supporting shared genetic variants underlying both *PARP14* and IBD susceptibility (Figure 8B). This genetic evidence, combined with its upregulation in active phase, highlights *PARP14* as a key driver of UC pathogenesis.

### 3.8. Immune Cell Infiltration Patterns Associated with UC Activity

**Figure 9:**
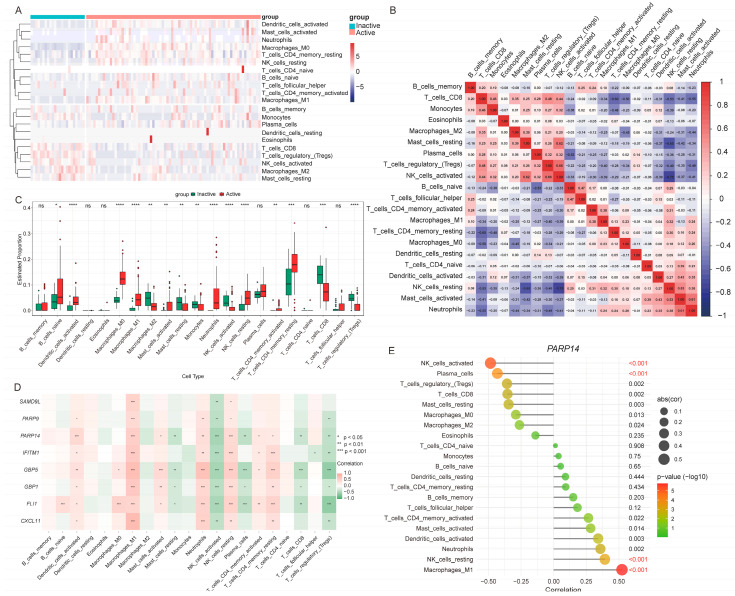
Immune infiltration patterns and correlations with characteristic genes in the training cohort. (**A**) Heatmap of immune cell infiltration abundances. (**B**) Correlation heatmap among immune cell subsets. (**C**) Box plots showing differential infiltration levels of immune cell subsets between inactive and active phase. (**D**) Correlation heatmap between characteristic genes and immune cell subtypes. (**E**) Lollipop plot for correlations between *PARP14* and immune cell subsets. Statistical significance is denoted as follows: ns, not significant; *, *p* < 0.05; **, *p* < 0.01; ***, *p* < 0.001; ****, *p* < 0.0001.

The immunological microenvironment was characterized using the LM22 signature matrix and GSE75214 transcriptome data. Relative abundances of 22 immune cell types were estimated with CIBERSORT (Figure 9A), while correlations among immune cells were assessed (Figure 9B). Several immune subsets displayed significant differences between inactive and active phase (Figure 9C) and correlated with characteristic genes (Figure 9D). Specifically, activated dendritic cells, M0 macrophages, M1 macrophages, neutrophils, resting NK cells, and resting CD4^+^ memory T cells were enriched in active phase and positively correlated with most characteristic genes. These increases align with the pro-inflammatory state of UC, implicating them in cytokine production and tissue injury. Conversely, M2 macrophages, activated NK cells, CD8^+^ T cells, and regulatory T cells (Tregs) were decreased and negatively correlated with the same genes, supporting consistent immune–molecular relationships. Notably, *PARP14* exhibited its most significant positive correlation with M1 macrophages and negative correlation with activated NK cells, further substantiating its potential role in modulating the immune imbalance associated with UC activity (Figure 9E).

Together, these results indicate that UC activity involves infiltration of pro-inflammatory immune cells, coupled with a decline in regulatory cell subsets. Moreover, significant molecular correlations suggest that *PARP14* and *FLI1* play pivotal roles in driving mucosal inflammation.

## 4. Discussion

The objective of this study was to identify potential molecular signatures of UC activity and elucidate their pathogenic roles by integrating transcriptomic analysis, machine learning, and genetic causal inference. To this end, a comprehensive analysis of 3 independent GEO datasets (GSE75214, GSE53306, GSE179285) was conducted, and the “Meorange” module was identified using WGCNA (R = 0.84, *p* < 0.05). This module demonstrated a robust correlation with UC activity. The integration of DEG analysis resulted in the identification of 138 core genes. Subsequent PPI networking and ensemble machine learning (RF + Stepglm [forward], average AUC = 0.877) prioritized 8 characteristic genes. Among these, *CXCL11*, *PARP14*, and *IFITM1* emerged as hub genes with exceptional diagnostic performance (average AUC: 0.855, 0.840, and 0.830, respectively). Furthermore, *FLI1* was identified as a pivotal transcription factor (TF) that regulates these hubs, and a causal relationship between *PARP14* upregulation and UC susceptibility was confirmed via SMR. These findings contribute to our enhanced understanding of the molecular and immunological mechanisms underlying the pathogenesis of UC.

A critical contribution of this study is the identification of *CXCL11*, *PARP14*, and *IFITM1* as stable diagnostic markers for UC activity, which outperform many previously reported UC biomarkers. For instance, *CCL11* and *MMP1* were identified as inflammation-related diagnostic biomarkers for UC, with AUC values of 0.741 and 0.703, respectively, in the GSE193677 dataset [18]. Eight novel biomarkers (e.g., TFF3, LRG, and HMGB1) were similarly evaluated [17]. In contrast, our hub genes (*CXCL11*, *PARP14*, and *IFITM1*) achieved consistently high average AUC values (0.830–0.855) across 3 independent cohorts of diverse ethnic backgrounds. Furthermore, SMR analysis confirmed a causal association between *PARP14* and UC susceptibility. The HEIDI *p*-value > 0.05 indicates that the genetic instrumental variables (SNPs) used in this study predominantly influence the outcome via the target exposure, rather than through irrelevant pathways—thus ruling out significant horizontal pleiotropy. This finding provides genetic evidence supporting the pathogenic role of *PARP14*. Collectively, these characteristics highlight *PARP14* as a potential candidate for clinical translation.

Active phase is characterized by marked dysregulation of immune cell infiltration, with pro-inflammatory subsets dominating the intestinal mucosal microenvironment [32,33]. This finding aligns with and extends the current understanding of UC immunopathogenesis. In our analysis, *PARP14* was significantly positively correlated with M1 macrophage infiltration and neutrophil accumulation—2 cell types strongly implicated in UC inflammation [34,35]. *PARP14* has been associated with the progression of inflammatory diseases by regulating Th2/Th17 signaling. It also acts as a downstream molecule of Klf5 involved in metabolic reprogramming and the polarization of microglia into the M1/M2 state [36,37]. These findings indicate that *PARP14* may play a key role in coordinating multiple pro-inflammatory processes in UC activity, including immune infiltration, inflammatory signaling, and cellular polarization.

Furthermore, *FLI1*, as a key TF regulating *CXCL11*, *PARP14*, and *IFITM1*, indirectly shapes immune infiltration: *CXCL11* (a CXC chemokine) recruits activated T cells and dendritic cells to the inflamed mucosa [18], while *IFITM1* activates the NF-κB pathway (by interacting with IKKβ to promote its phosphorylation) to regulate immune and inflammatory responses [38]. Consistent with these findings, our immune cell deconvolution analysis indicated that *FLI1* expression was positively correlated with Th17 cell infiltration—a subset known to drive UC activity via IL-17 secretion [39]. These data collectively demonstrate that *FLI1* orchestrates a pro-inflammatory transcriptional program, amplifying immune cell infiltration through *PARP14* and downstream genes, ultimately exacerbating intestinal mucosal damage.

A methodological consideration in this study is the use of IBD GWAS data rather than UC activity-specific GWAS data for SMR analysis. This approach was necessitated by current limitations in available genomic resources. To date, most public GWAS datasets for UC focus on disease susceptibility [39,40], with few explicitly stratifying samples by UC activity. Therefore, the IBD GWAS data were integrated with eQTL data from intestinal mucosal tissues [7]. While this approach cannot capture activity-specific genetic effects, it nonetheless establishes a causal relationship between *PARP14* and UC susceptibility (rather than just UC activity), providing critical genetic validation for our transcriptomic findings. Future studies should prioritize generating activity-stratified UC activity GWAS and eQTL data to refine causal inferences and identify activity-specific genetic regulators.

A key finding of this study is the identification of the *FLI1-PARP14* axis as a central regulator of UC activity, with implications for long-term complications such as colorectal cancer (CRC). Chronic intestinal inflammation is a well-established driver of CRC in UC patients [41], and our data suggest that the *FLI1-PARP14* axis may contribute to this progression: (1) *FLI1* regulates *PARP14* and downstream pro-inflammatory genes, sustaining a chronic inflammatory microenvironment that promotes DNA damage accumulation in intestinal epithelial cells by increasing reactive oxygen species (ROS) and impairing DNA repair [34,35]; (2) *PARP14*-mediated immune infiltration (e.g., M1 macrophages, neutrophils) further amplifies inflammation-associated tissue damage, creating a self-reinforcing loop that accelerates epithelial dysplasia [41]; (3) *FLI1* itself has been implicated in cancer progression by regulating cell proliferation and epithelial–mesenchymal transition [35], suggesting that it may directly and indirectly contribute to CRC development in long-standing UC. While functional experiments are needed to confirm these links, our data provide a novel framework: targeting the *FLI1-PARP14* axis could not only alleviate UC activity by reducing immune infiltration but also potentially lower CRC risk by mitigating chronic inflammation.

Several limitations of this study warrant consideration. First, reliance on GEO datasets introduces potential selection bias (e.g., overrepresentation of patients of European ancestry), which may limit the generalizability of our hub genes to diverse populations. Second, transcriptomic data alone cannot confirm protein-level expression; future studies should validate *PARP14* in UC mucosal biopsies via immunohistochemistry or Western blotting [18]. Third, functional experiments (e.g., *PARP14* knockdown in intestinal epithelial cells, *FLI1* ChIP-seq to confirm binding to the *PARP14* promoter) are needed to directly verify the regulatory mechanisms identified. Fourth, potential crosstalk between *PARP14* and gut microbiota—an important factor in UC pathogenesis was not explored [6]—which may further clarify its role in inflammation. To address these gaps, future research could: (1) validate hub genes in multi-ethnic cohorts and explore non-invasive detection (e.g., serum or fecal *PARP14*); (2) test *PARP14* inhibitors (e.g., RBN012759) in dextran sulfate sodium (DSS)-induced UC mice to evaluate effects on mucosal healing and CRC development [42]; (3) integrate microbiomics data to investigate interactions between *PARP14* and gut microbiota [6].

## 5. Conclusions

In this study, *PARP14* was identified as a potential biomarker associated with UC activity, demonstrating promising clinical utility. A potential *FLI1–PARP14*–immune infiltration axis was further proposed, which may be involved in UC pathogenesis: FLI1 may be associated with *PARP14* and downstream genes, potentially leading to increased infiltration of M1 macrophages, neutrophils, and Th17 cells, thereby suggested to exacerbate intestinal mucosal inflammation. This axis is also proposed to be linked to the potential risk of CRC in chronic UC, possibly through the maintenance of inflammatory damage and influence on epithelial dysplasia. These findings are considered to provide insights into the molecular mechanisms of UC and to lay a foundation for further research on precision medicine approaches for UC diagnosis and treatment.

## Figures and Tables

**Table 1:** GEO datasets and samples.

Datasets	Samples from UC Patients		Groups
	Inactive Phase	Active Phase	
GSE75214	23	74	Training cohort
GSE53306	12	16	Testing cohorts
GSE179285	32	23	

**Table 2:** AUC of 8 characteristic genes in the training cohort, and testing cohorts.

Gene	Training	Testing		Average AUC
	GSE75214	GSE53306	GSE179285	
*CXCL11*	0.9	0.771	0.894	0.855
*PARP14*	0.952	0.703	0.864	0.840
*IFITM1*	0.973	0.729	0.789	0.830
*SAMD9L*	0.975	0.562	0.874	0.804
*GBP1*	0.949	0.646	0.792	0.796
*GBP5*	0.961	0.609	0.803	0.791
*PARP9*	0.936	0.604	0.818	0.786
*CXCL10*	0.907	0.609	0.764	0.760

**Table 3:** Transcription factors (TFs) targeting characteristic genes.

Key TF	Description	Training		GSE53306		GSE179285	
		*p* Value	log2FC	*p* Value	log2FC	*p* Value	log2FC
*FLI1*	Fli-1 proto-oncogene, ETS transcription factor	1.07 × 10^−10^	1.078	0.0305	0.560	9.47 × 10^−4^	0.702
*STAT1*	signal transducer and activator of transcription 1	3.87 × 10^−14^	1.035	0.404	−0.194	1.96 × 10^−4^	0.833
*IRF4*	interferon regulatory factor 4	4.67 × 10^−16^	1.665	0.162	0.453	7.27 × 10^−5^	0.739

**Table 4:** *PARP14* SMR analysis.

Gene	b_SMR	se_SMR	p_SMR	p_HEIDI	nsnp_HEIDI
*PARP14*	0.0722	0.0348	0.0378	0.240	20

## Data Availability

The RNA expression dataset is publicly accessible through the NCBI GEO repository at https://www.ncbi.nlm.nih.gov/geo/ (accessed on 5 August 2025). All findings from this research have been incorporated into the manuscript. Additional data can be obtained by contacting the corresponding author with a formal request.

## Abbreviations

The following abbreviations are used in this manuscript:

AUC	Area under the receiver operating characteristic curve
CD	Crohn’s disease
CRC	Colorectal cancer
DEG	Differentially expressed gene
DSS	Dextran sulfate sodium
eQTL	Expression quantitative trait locus
GBM	Gradient boosting machine
GO	Gene ontology
GWAS	Genome-wide association study
IBD	Inflammatory bowel disease
KEGG	Kyoto encyclopedia of genes and genomes
LDA	Linear discriminant analysis
LM22	Leukocyte signature matrix
Lasso	Least absolute shrinkage and selection operator
MCC	Maximal clique centrality
PPI	Protein–protein interaction
plsR-glm	Partial least squares regression with generalized linear model
Ridge	Ridge regression
RF	Random forest
ROC	Receiver operating characteristic
SMR	Summary-data–based mendelian randomization
SVM	Support vector machine
Stepglm	Stepwise generalized linear model
glmBoost	Boosted generalized linear model
UC	Ulcerative colitis
WGCNA	Weighted gene co-expression network analysis
XGBoost	Extreme gradient boosting
